# Supplementary material for: Methyl Salicylate Increases Attraction and Function of Beneficial Arthropods in Cranberries
Source: Insects. 2019 Nov 25;10(12):423. doi: 10.3390/insects10120423 (PMC6955811; doi:10.3390/insects10120423)
Supplement: Supplementary file 1 [file insects-10-00423-s001.pdf]

Methyl Salicylate Increases Attraction and Function of Beneficial Arthropods in Cranberries

Jordano Salamanca, Brígida Souza, Vera Kyryczenko-Roth and Cesar Rodriguez-Saona

Supplementary material

Table S1. Number of insecticide applications throughout the 2011 and 2012 seasons in cranberry bogs.

Year	Farm	Bog	Application Date	Active Ingredient	Trade Name	Rate (per Acre)
2011	Farm A	1	14-Jun	Chlorpyrifos	Warhawk	3 pts
		2		Chlorpyrifos	Warhawk	3 pts
		3		Chlorpyrifos	Warhawk	3 pts
		4		Chlorpyrifos	Warhawk	3 pts
	Farm B	1	11-May	Spinetoram	Delegate WG	6 oz
		2	12-May	Methoxyfenozide	Intrepid 2F	16 oz
			28-May	Chlorpyrifos	Lorsban 4E	1.5 pts
		3	-	-	-	-
		4	-	-	-	-
	Farm C	1	11-Jul	Imidacloprid	Admire Pro	14 oz
		2		Imidacloprid	Admire Pro	14 oz
		3		Imidacloprid	Admire Pro	14 oz
		4		Imidacloprid	Admire Pro	14 oz
	Farm D	1	14-May	Methoxyfenozide	Intrepid 2F	16 oz
			26-May	Diazinon	Diazinon AG600 WBC	2 qt
			11-Jul	Diazinon	Diazinon AG600 WBC	2 qt
		2	14-May	Methoxyfenozide	Intrepid 2F	16 oz
			26-May	Diazinon	Diazinon AG600 WBC	2 qt
			11-Jul	Diazinon	Diazinon AG600 WBC	2 qt
		3	26-May	Diazinon	Diazinon AG600 WBC	2 qt
			11-Jul	Diazinon	Diazinon AG600 WBC	2 qt
		4	26-May	Diazinon	Diazinon AG600 WBC	2 qt
			11-Jul	Diazinon	Diazinon AG600 WBC	2 qt
2012	Farm A	1	16-Jul	Diazinon	Diazinon AG500	2 qt
		2		Diazinon	Diazinon AG500	2 qt
		3		Methoxyfenozide	Intrepid 2F	1 pt
		4		Diazinon	Diazinon AG500	2 qt
	Farm B	1	18-May	Chlorpyrifos	Warhawk	1.5 pt
		2	25-May	Methoxyfenozide	Intrepid 2F	16 floz
		3	23-May	Methoxyfenozide	Intrepid 2F	16 floz
		4	-	-	-	-
		1	19-May	Methoxyfenozide	Intrepid 2F	16 floz

	Farm C		10-Jul	Diazinon	Diazinon AG500	3 qt
			25-Aug	Diazinon	Diazinon AG500	2 qt
		2	19-May	Methoxyfenozide	Intrepid 2F	16 floz
			10-Jul	Diazinon	Diazinon AG500	3 qt
		3	19-May	Methoxyfenozide	Intrepid 2F	16 floz
			10-Jul	Diazinon	Diazinon AG500	3 qt
		4	19-May	Methoxyfenozide	Intrepid 2F	16 floz
			10-Jul	Diazinon	Diazinon AG500	3 qt
			25-Aug	Diazinon	Diazinon AG500	2 qt
	Farm D	1	31-May	Methoxyfenozide	Intrepid 2F	16 floz
			09-Jul	Diazinon	Diazinon AG600 WBC	2 qt
		2	31-May	Methoxyfenozide	Intrepid 2F	16 floz
			09-Jul	Diazinon	Diazinon AG600 WBC	2 qt
		3	31-May	Methoxyfenozide	Intrepid 2F	16 floz
			09-Jul	Diazinon	Diazinon AG600 WBC	2 qt
		4	31-May	Methoxyfenozide	Intrepid 2F	16 floz
			09-Jul	Diazinon	Diazinon AG600 WBC	2 qt
